# Gas sensing properties of conducting polymer/Au-loaded ZnO nanoparticle composite materials at room temperature

**DOI:** 10.1186/1556-276X-9-467

**Published:** 2014-09-04

**Authors:** Viruntachar Kruefu, Anurat Wisitsoraat, Adisorn Tuantranont, Sukon Phanichphant

**Affiliations:** 1 Program in Materials Science, Faculty of Science, Maejo University, Chiang Mai 50290, Thailand; 2 National Electronics and Computer Technology Center, Pathumthani 12120, Thailand; 3 Materials Science Research Center, Faculty of Science, Chiang Mai University, Chiang Mai 50200, Thailand

**Keywords:** P3HT, Au-loaded ZnO, Composite films, NH_3_ sensor, Flame spray pyrolysis

## Abstract

In this work, a new poly (3-hexylthiophene):1.00 mol% Au-loaded zinc oxide nanoparticles (P3HT:Au/ZnO NPs) hybrid sensor is developed and systematically studied for ammonia sensing applications. The 1.00 mol% Au/ZnO NPs were synthesized by a one-step flame spray pyrolysis (FSP) process and mixed with P3HT at different mixing ratios (1:1, 2:1, 3:1, 4:1, and 1:2) before drop casting on an Al_2_O_3_ substrate with interdigitated gold electrodes to form thick film sensors. Particle characterizations by X-ray diffraction (XRD), nitrogen adsorption analysis, and high-resolution transmission electron microscopy (HR-TEM) showed highly crystalline ZnO nanoparticles (5 to 15 nm) loaded with ultrafine Au nanoparticles (1 to 2 nm). Film characterizations by XRD, field-emission scanning electron microscopy (FE-SEM), energy-dispersive X-ray (EDX) spectroscopy, and atomic force microscopy (AFM) revealed the presence of P3HT/ZnO mixed phases and porous nanoparticle structures in the composite thick film. The gas sensing properties of P3HT:1.00 mol% Au/ZnO NPs composite sensors were studied for reducing and oxidizing gases (NH_3_, C_2_H_5_OH, CO, H_2_S, NO_2_, and H_2_O) at room temperature. It was found that the composite film with 4:1 of P3HT:1.00 mol% Au/ZnO NPs exhibited the best NH_3_ sensing performances with high response (approximately 32 to 1,000 ppm of NH_3_), fast response time (4.2 s), and high selectivity at room temperature. Plausible mechanisms explaining the enhanced NH_3_ response by composite films were discussed.

## Background

Gas sensors for ammonia (NH_3_) detection at low concentration are of great scientific importance in environmental monitoring, medical diagnosis, and various chemical/agricultural industries, since ammonia is very harmful to humans and the environment [1-5]. Several semiconducting metal oxides are highly promising for NH_3_ detection due to their excellent response [6-8]. However, they suffer from some inconvenience including high operating temperatures (200°C to 400°C) [6-11]. High operating temperature results in high power consumption and complicated sensor design/fabrication [12]. Thus, ammonia sensors operable at room temperature with long life time are of great interest.

Conducting polymers, such as polypyrrole (PPy), polyaniline (Pani), polythiophene (PTh), and their derivatives, have demonstrated gas sensing capability at low or even room temperature [13,14]. However, they are still not practically useful due to comparatively low response, lack of specificity, and relatively poor stability. A summary of gas sensing properties of NH_3_ gas sensor-based conducting polymers as well as their hybrids prepared by various methods is shown in Table 1. Firstly, polyaniline (PANI) nanowires (NWs) prepared by photolithography process are shown to exhibit a high sensor response of approximately 3 at 500 ppm NH_3_ in synthetic air [14]. In addition, surface acoustic wave (SAW) NH_3_ gas sensors based on PPy prepared by layer-by-layer (LBL) self-assembly method are investigated for NH_3_ sensing with different numbers of layer. The sensor with two layers of PPy shows the best performance relative to those with other numbers of PPy layers [15]. Additionally, NH_3_ gas sensors based on organic thin-film transistors (OTFTs) made from spin-coated poly (3-hexylthiophene) (P3HT) on a thermally grown SiO_2_/Si wafer exhibit a sensor response of 0.31 to 100 ppm NH_3_ at room temperature [16]. Among these, P3HT is particularly promising for gas sensing applications due to its selective room-temperature response toward some gases especially ammonia and NO_2_[16-18] and its relatively high stability. P3HT is known to have high oxidation potential making it highly stable in doped/undoped states under ambient conditions at room temperature and has specific chemical interactions with some gases [17].

**Table 1:** **Summary of NH**_**3 **_**sensing properties of a conducting polymer and metal or metal oxide/conducting polymer sensor**

**Authors/reference**	**Method**	**Materials**	**NH**_**3 **_**concentration (ppm)**	**NH**_**3 **_**sensing performances**
Chen et al. [15]	Layer-by-layer (LBL) self-assembly method	Polypyrrole (PPy) and Pt-doped two-layer PPy thin films	100	Response: approximately 3 to 100 ppm NH_3_ at room temperature
Jeong et al. [16]	Spin coating	P3HT thin-film transistors	10 to 100	Response: 0.31 to 100 ppm NH_3_ at room temperature
Saxena et al. [27]	Drop casting	P3HT:ZnO nanowire thin films	4	Response: <1% to 4 ppm NH_3_ at room temperature
Chougule et al. [13]	Low-frequency AC spin coating	CSA (30 wt.%) doped PPy-ZnO hybrid films	100	Response: approximately 11 to 100 ppm NH_3_ at room temperature
Baratto [18]	Drop casting	Hybrid poly (3-hexylthiophene)-ZnO nanocomposite thin films	25	Response: small response to 25 ppm NH_3_ at room temperature
Tuan et al. [14]	A standard photolithography technique	Polyaniline (PANI) nanowires (NWs)	25 to 500	Response: 2.9 to 500 ppm NH_3_ at room temperature
Tai et al. [21]	*In situ* self-assembly	Polyaniline/titanium dioxide (PANI/TiO_2_) nanocomposite thin films	23 to 141	Response: approximately 9 to 140 ppm NH_3_, response time 2 s, and recovery time 20 to 60 s at room temperature
Huang et al. [26]	Spin coating	Graphene oxide (RGO)-polyaniline (PANI) hybrids	50	Response: approximately 10.4 to 50 ppm NH_3_ at room temperature
Dhingra et al. [23]	Dipping	Zinc oxide*/*polyaniline (ZnO*/*PANI) hybrid	300	Response: approximately 23 to 300 ppm NH_3_ at room temperature
This work	Drop casting	P3HT:1.00 mol% Au/ZnO NPs (4:1)	50 to 1,000	Response: approximately 32 to 1,000 ppm NH_3_ at room temperature

The advantages of organic materials can be further exploited by their combinations with metal oxides [13,18-23] and metals [15,19,24,25]. For instance, the hybrid of graphene oxide (GO)-polyaniline (PANI) nanoparticles prepared by spin coating is shown to exhibit higher response to 50 ppm of NH_3_ than those of bare PANI nanofiber and bare GO sensors by a factor of 3.4 and 10.4, respectively [26]. In addition, P3HT/ZnO NWs and polypyrrole-zinc oxide (PPy-ZnO) composites are reported for sensitive detection of NH_3_[13,27]. In contrast, another report of P3HT-ZnO NW thin films demonstrates high sensitivity for NO_2_ or H_2_S and a moderate sensitivity for CO [27], while the response to NH_3_ was very low (*S <* 1%) at room temperature. Furthermore, PPy-ZnO hybrid films are doped with camphor sulfonic acid (30 wt.%) and exhibit high selectivity to NO_2_, high sensitivity at low NO_2_ concentration (80% to 100 ppm), fast response time (120 s), and good stability but relatively sluggish response to reducing gases (H_2_S, NH_3_, C_2_H_5_OH, and CH_3_OH) at room temperature [13]. Moreover, novel P3HT-ZnO nanocomposite hybrid thin films show a high relative response of 2.2 to 200 ppb of NO_2_ but virtually no response to CO or C_2_H_5_OH and very small response to NH_3_ at room temperature [18]. Besides, zinc oxide*/*polyaniline (ZnO*/*PANI) hybrid structures are confirmed to exhibit much higher sensitivity to NH_3_ gas at room temperature than bare ZnO [23,28].

It can be observed that ZnO nanostructures are among the most widely employed metal oxides in polymer-based hybrid gas sensors, which should be due to its observed gas sensing enhancement, abundance, low cost, high stability, high electron mobility, low crystallization temperature, and ease of fabrication. However, mechanisms for gas sensing enhancement provided by ZnO nanostructures are not yet well understood. Nevertheless, it is widely observed that sensing properties of the hybrid sensors are related to surface characteristics of ZnO, which significantly depend on fabrication processes [29]. Most reported work mostly employs chemical-route and chemical vapor deposition (CVD) methods, which suffer from either poor reproducibility or high cost. Alternative low-cost, effective, and reliable methods for mass production of metal oxide nanostructured components in composite are still needed. Flame spray pyrolysis (FSP) is one of the most promising routes for the formation of single and multi-component functional nanoparticles with well-controlled diameter at low cost and high production rate. FSP has been applied to prepare metal oxide-supported nanoparticles and heterogeneous catalysts. However, FSP-made materials have not been employed in polymer-metal oxide hybrid sensors. It is thus interesting to apply them in this sensor system.

Gold (Au) is another effective means to improve sensing performance of polymer-based gas sensors via catalytic effects, which may be attained at low or room temperature. For instance, Pd incorporation in PANI considerably improved the response to methanol [19]. Similarly, Pt loading in PPy gas-sensitive films considerably improved NH_3_ responses of the PPy sensor [15]. Au is another effective catalyst for gas sensing [30]. Au loading on metal oxides particularly ZnO nanostructures has demonstrated to enhance response toward C_2_H_5_OH [31] and CO [30]. However, Au is relatively much less employed in polymer-based hybrid gas sensors. Its effect on gas sensing of a polymer-based hybrid sensor should thus be investigated.

Furthermore, the combination of noble metal catalyst, metal oxide, and polymer is expected to offer superior room-temperature gas sensors. To date, there has been development of noble metal/metal oxide/polymer composite gas sensors. In this work, we propose a practical implementation of this approach by blending a P3HT conductive polymer with Au-loaded ZnO nanoparticles (NPs) prepared by FSP. The novel hybrid materials are structurally characterized and tested for ammonia detection. In addition, the effects of ZnO and gold loading on gas sensing properties of P3HT sensing films are systematically analyzed by comparing the performances of P3HT with and without unloaded and 1.00 mol% Au-loaded ZnO NPs.

## Methods

### Synthesis and characterization of nanoparticles

**Figure 1:**
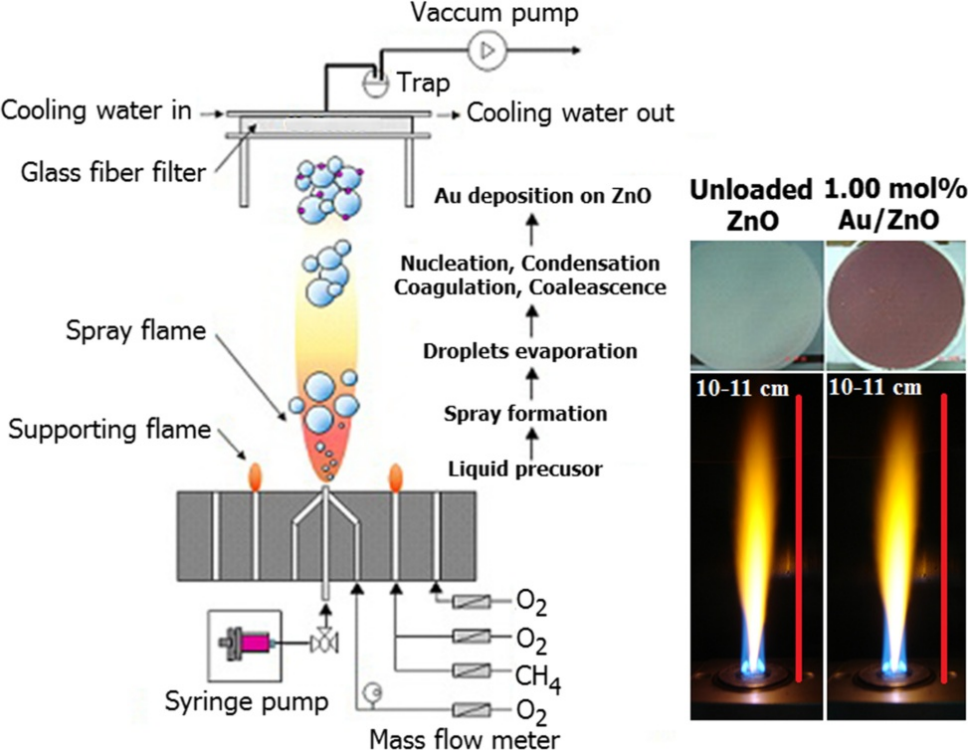
The experimental setup for flame-made unloaded ZnO and 1.00 mol% Au/ZnO NPs.

The 1.00 mol% Au-loaded ZnO nanoparticles (Au/ZnO NP_S_) were successfully synthesized by the FSP process schematically illustrated in Figure 1. The precursor solution for FSP was prepared from zinc naphthenate (Sigma-Aldrich, St. Louis, MO, USA; 8 wt.% Zn) and gold (III)-chloride hydrate (Sigma-Aldrich; ≥49% Au) diluted in ethanol (Carlo Erba Reagenti SpA, Rodano, Italy; 98.5%). The precursor solution was injected at 5 mL min^-1^ through the reactor nozzle and dispersed with 5.0 L min^-1^ of oxygen into a fine spray (5/5 flame) while maintaining a constant pressure drop of 1.5 bar across the nozzle tip. A premixed flame fueled by 1.19 L min^-1^ of methane and 2.46 L min^-1^ of oxygen was ignited and maintained to support the combustion of the spray. The flames have yellowish orange color with a height of approximately 10 to 11 cm for both unloaded ZnO and 1.00 mol% Au/ZnO as shown in Figure 1.

Upon evaporation and combustion of precursor droplets, particles are formed by nucleation, condensation, coagulation, coalescence, and Au deposition on a ZnO support. Finally, the nanoparticles were collected from glass microfiber filters (Whatmann GF/D, 25.7 cm in diameter) placed above the flame with an aid of a vacuum pump. X-ray diffraction (TTRAXIII diffractometer, Rigaku Corporation, Tokyo, Japan) was employed to confirm the phase and crystallinity of obtained nanoparticles using CuKα radiation at 2*θ* = 20° to 80° with a step size of 0.06° and a scanning speed of 0.72°/min. Brunauer-Emmett-Teller (BET) analysis by nitrogen absorption (Micromeritics Tristar 3000, Micromeritics Instrument Co., Norcross, GA, USA) at liquid nitrogen temperature (77.4 K) was performed to obtain the specific surface area of the nanoparticles. The average particle size (*d*_BET_) in nanometers is equal to 6/(SSA_BET_ × *ρ*_av_), where SSA_BET_ is the specific surface area (m^2^/g) and *ρ*_av_ is the average density of the flame-made 1.00 mol% Au/ZnO NPs with *ρ*_ZnO_ = 5.606 g cm^-3^[32,33] and *ρ*_Au_ = 19.32 g cm^-3^[24], which took into account their weight content. High-resolution transmission electron microscopy (HR-TEM) was employed to examine the morphology and size of nanoparticles. The elemental composition of nanoparticles was analyzed by energy-dispersive X-ray spectroscopy (EDX) in mapping mode to confirm Au content in the resultant powders.

### Sensor fabrication and sensing film characterization

Composite sensors were prepared by blending P3HT (Rieke Metals, Inc., Lincoln, NE, USA; *M*_*w*_ 48,000 g mol^-1^) solution with 1.00 mol% Au/ZnO NP colloidal solution and drop casting onto prefabricated Cr/Au interdigitated electrodes. Cr (50 nm thick) and Au (200 nm thick) layers were deposited by DC sputtering in argon gas at a pressure of 3 × 10^-3^ mbar on an alumina substrate (0.40 cm × 0.55 cm × 0.04 cm). The interdigit spacing, width, and length were 100 μm, 100 μm, and 0.24 cm, respectively. P3HT solution was prepared by dissolving 30 mg of P3HT in 0.50 mL of chlorobenzene, and Au/ZnO NP colloidal solution was made by dispersing 5 to 25 mg of ZnO nanoparticles (unloaded ZnO and 1.00 mol% Au/ZnO) in 0.50 mL of 1-butanol.

To prepared hybrid films with various compositions, 1.00 mol% Au/ZnO NP colloidal solution was added to the stirred P3HT solution with five different mixing ratios (1:1, 2:1, 3:1, 4:1, and 1:2). The blended solution was drop casted on the interdigitated electrode and then baked at 150°C for 3 min in an oven. The active area of these sensing devices is 0.12 ± 0.04 cm^2^. After completion, the crystalline phase of composite films was characterized by X-ray diffraction (XRD). The surface morphologies, elemental analysis, and cross section of the sensing layers were verified by field-emission scanning electron microscopy (FE-SEM) equipped with an EDX analysis system. Finally, the devices were transferred to a stainless steel chamber for gas sensing measurement at room temperature.

### Electrical and sensing test

P3HT and P3HT:1.00 mol% Au/ZnO NPs sensors were then tested by the standard flow through method in a stainless steel chamber at room temperature (25°C). The sensing experiment was carried out by measuring the reversible change of electrical resistance of sensors taken through a 6517 Keithley resistance meter (Keithley Instruments Inc., Cleveland, OH, USA) under a DC applied voltage of 10 V. A constant flux of synthetic dry air of 1 L/min as gas carrier was flowed to mix with the desired concentration of pollutants dispersed in synthetic air, and gas flow rates were precisely manipulated using a computer-controlled multi-channel mass flow controller. The background relative humidity (RH) under a flux of dry air was measured to be around 10%. The NH_3_ pollutant source is a calibrated ammonia vapor balanced in dry air at 4,000 ppm (Linde Co. Ltd, Bangkok, Thailand). Ammonia (NH_3_) vapor concentration was varied from 25 to 1,000 ppm. The gas sample exposure time and the dry air restoring time were fixed at 10 and 25 min, respectively. For selectivity performance, the sensors were also tested toward C_2_H_5_OH, CO, H_2_S, and NO_2_ at 1,000 ppm. The effect of humidity was also tested at 80% RH.

## Results and discussion

### Particles and sensing film properties

**Figure 2:**
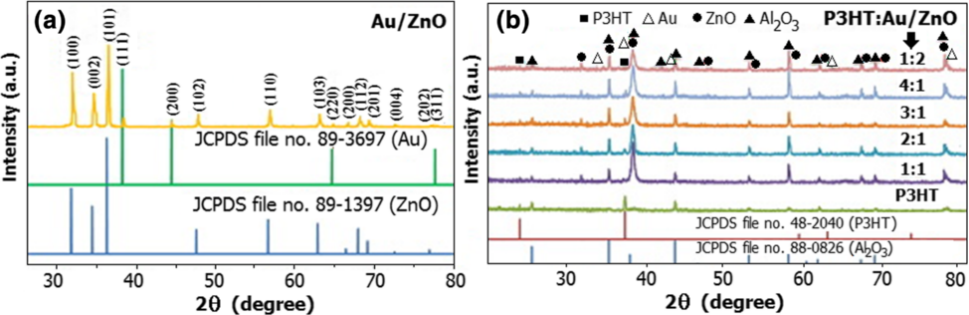
**XRD patterns. (a)** 1.00 mol% Au/ZnO NPs. **(b)** Sensing films of P3HT:1.00 mol% Au/ZnO NPs in difference ratio.

The XRD pattern of 1.00 mol% Au/ZnO NPs as shown in Figure 2a reveals that the nanoparticle is highly crystalline and has the hexagonal structure of ZnO according to JCPDS no. 89–1397. Au peaks are also found in these patterns and well matched with a face-centered cubic phase of Au (JCPDS file no. 89–3697 [34]). The XRD patterns of P3HT and P3HT:1.00 mol% Au/ZnO NPs composite sensing films coated on Au/Al_2_O_3_ substrates in Figure 2b indicate the presence of the P3HT monoclinic crystal (the JCPDS no. 48–2040), the hexagonal ZnO phase of the NPs, a fcc phase of Au (JCPDS file no. 89–3697 [34]), and a corundum phase of Al_2_O_3_ (JCPDS file no. 88–0826 [35]). It can be seen that Au peaks of the hybrid film are relatively pronounced compared with those of 1.00 mol% Au/ZnO NPs. These observed Au peaks are mainly attributed to the diffraction from the interdigitated Au electrode, which almost completely overrides the very weak diffraction from Au loaded on ZnO NPs.

**Figure 3:**
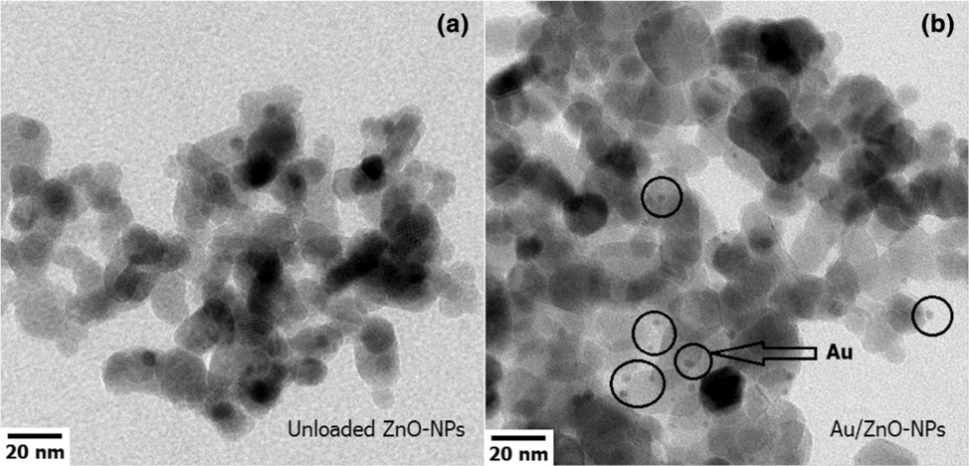
**HR-TEM bright-field image. (a)** Unloaded ZnO. **(b)** 1.00 mol% Au/ZnO NPs.

The specific surface area of the unloaded ZnO and 1.00 mol% Au/ZnO NPs was measured by nitrogen absorption using BET analysis. It was found that the specific surface area (SSA_BET_) of unloaded ZnO and 1.00 mol% Au/ZnO NPs is about 86.3 and 100 m^2^ g^-1^, respectively. The corresponding BET equivalent particle diameters (*d*_BET_) of unloaded ZnO and 1.00 mol% Au/ZnO NPs are calculated to be about 10 and 9 nm, respectively. Thus, 1.00 mol% Au loading on ZnO NPs increases the specific surface area by 15% and reduces the particle diameter by about 10%. HR-TEM images of unloaded ZnO and 1.00 mol% Au/ZnO NPs in Figure 3 show spherical nanoparticles along with a few nanorods having a size in the range of 5 to 15 nm. For Au-loaded ZnO (Figure 3b), smaller spherical NPs with an average diameter of approximately 1.5 nm are clearly observed on the surface of ZnO as the darker spots as indicated in the figure. These NPs are confirmed to be Au NPs on ZnO support by EDX analysis in mapping mode (data not shown). The observed particle diameters by HR-TEM are in the same range as BET data. The observed smaller Au nanoparticle diameter of approximately 1.5 explains the result that the average BET nanoparticle diameter becomes smaller with Au loading as the average particle size will be reduced by the contribution of smaller particles.

**Figure 4:**
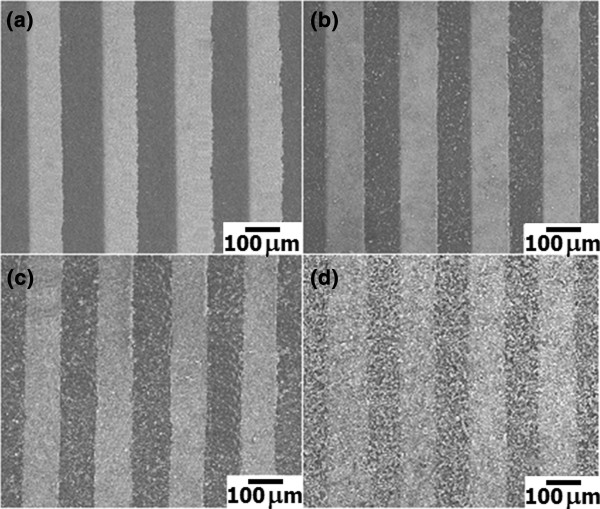
**FE-SEM images. (a)** P3HT. **(b-d)** P3HT:1.00 mol% Au/ZnO NPs sensing films with the mixing ratios of 3:1, 2:1, and 1:2, respectively, on an Al_2_O_3_ substrate with interdigitated Au electrodes.

Figure 4 shows FE-SEM images of P3HT and P3HT:1.00 mol% Au/ZnO NPs composite sensing films with the ratios of 4:1, 2:1, and 1:2 deposited on Al_2_O_3_ substrates with interdigitated Au electrodes. It can be seen that the surface of P3HT film is smooth and conformal with Au lines of the interdigitated electrode (Figure 4a). Upon mixing with 1.00 mol% Au/ZnO NPs, the surface becomes a relatively rough covering with fine white spots of NPs. The distribution of these spots on the Au interdigitated electrode surface is quite uniform, and the density of white spots increases accordingly with increasing content of NPs (Figure 4b, c, d). The results confirm the homogenous dispersion of 1.00 mol% Au/ZnO NPs in the P3HT matrix and its conformal coating on the substrate. In addition, the specific surface area of the composite film should be increased with increasing content of 1.00 mol% Au/ZnO NPs.

**Figure 5:**
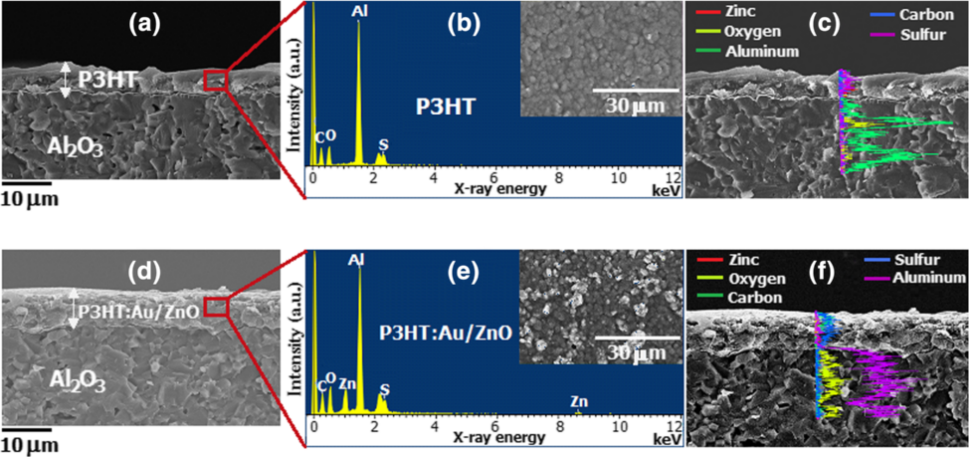
**FE-SEM micrographs of the cross-sectional structure. (a)** P3HT. **(d)** P3HT:1.00 mol% Au/ZnO NPs sensing films on an alumina substrate. **(b**, **e)** Corresponding EDX. **(c**, **f)** Corresponding line scan profiles.

The cross-sectional FE-SEM images along with EDX analyses of P3HT and P3HT:1.00 mol% Au/ZnO NPs (4:1) composite sensing films on an Al_2_O_3_ substrate with interdigitated Au electrodes after sensing test at room temperature in dry air are illustrated in Figure 5. It can be seen that the P3HT film is a smooth and solid layer (Figure 5a, b, c), while the composite film demonstrates porous asperities of the nanoparticle-polymer mixture (Figure 5d, e, f). The thicknesses of P3HT and composite films are estimated in the same range of 6 to 8 μm. The elemental composition on the surface and across P3HT and P3HT:1.00 mol% Au/ZnO NP layers is demonstrated in the EDX spectra and line scan profiles (Figure 5b, c and 5e, f, respectively). It confirms that the P3HT film contains only oxygen (O), carbon (C), and sulfur (S) and the P3HT:1.00 mol% Au/ZnO NP layer has one additional element of zinc (Zn) while the gold (Au) loaded element cannot be observed due to its very low content. In addition, the line scan profiles indicate that elemental compositions through the films are quite uniform.

**Figure 6:**
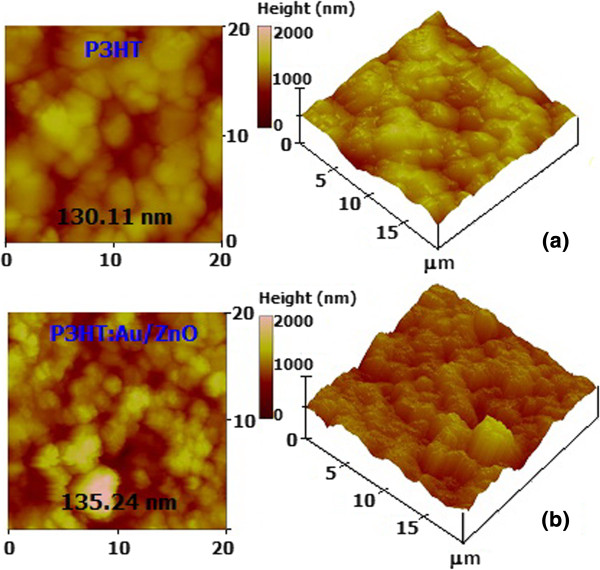
**AFM morphology. (a)** P3HT. **(b)** P3HT:1.00 mol% Au/ZnO NPs (4:1) hybridized sensing film drop casted on an Al_2_O_3_ substrate.

Atomic force microscopy (AFM) was employed to quantitatively investigate the morphology of P3HT and P3HT:1.00 mol% Au/ZnO NPs (4:1) composite sensing films drop casted on the Al_2_O_3_ substrate (Figure 6). The results indicate that the film surfaces are quite uniform, containing only tiny defects within a scan area of 20 μm × 20 μm. The average surface roughness of P3HT and the P3HT:1.00 mol% Au/ZnO NPs film is calculated from AFM data to be 130.1 and 135.2 nm, respectively. In addition, the composite film exhibits a relatively sharp granular morphology with a uniform grain size of approximately 80 to 100 nm, suggesting the presence of a nanosized grain structure in the composite sensing film due to the addition of 1.00 mol% Au/ZnO NPs.

### Gas sensing properties

**Figure 7:**
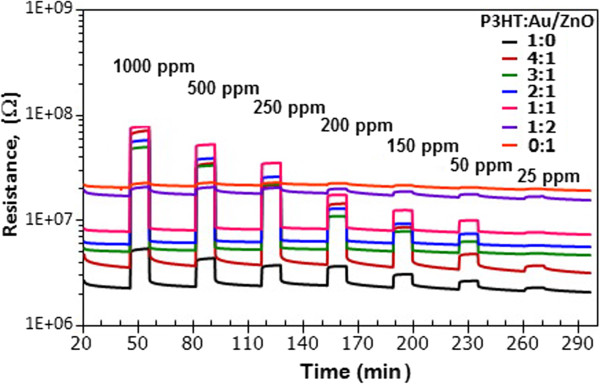
**Change in resistance.** The resistance of sensors with difference ratio of P3HT:1.00 mol% Au/ZnO NPs (1:0, 1:1, 2:1, 3:1, 4:1, 1:2, and 0:1) toward 25 to 1,000 ppm NH_3_ at room temperature.

The dynamic changes in resistance of sensors with different mixing ratios of P3HT:1.00 mol% Au/ZnO NPs (1:0, 1:1, 2:1, 3:1, 4:1, 1:2, and 0:1) are shown in Figure 7. It is seen that all sensors exhibit an increase of resistance during NH_3_ exposure, indicating a p-type-like gas sensing behavior. In addition, it is observed that the baseline resistance monotonically increases with increasing content of 1.00 mol% Au/ZnO NPs in accordance with the typical combination of materials’ resistances. Furthermore, P3HT exhibits a moderate NH_3_ response, while 1.00 mol% Au/ZnO NPs give very low response to NH_3_ at room temperature. Moreover, the addition of 1.00 mol% Au/ZnO NPs into P3HT at a mixing ratio up to 1:1 leads to significant enhancement in the NH_3_ response compared with the P3HT sensor. However, the response rapidly degrades when the amount of 1.00 mol% Au/ZnO NPs exceeds that of P3HT (1:2). From calculated changes of resistance, it is found that the sensor with 4:1 of P3HT:1.00 mol% Au/ZnO NPs exhibits the highest value, indicating that it is the optimal P3HT:1.00 mol% Au/ZnO NPs composite sensor. Since the optimal mixing ratio of the Au/ZnO NPs and P3HT of 1:4 is at the lowest border of the investigated range, it is possible that the actual optimal concentration will be at a lower concentration value and further detailed investigation should be conducted to refine the result. The obtained optimal performances of P3HT:Au/ZnO sensors are superior to other reports presented in Table 1 with a relatively high response magnitude of 32 and wide concentration range of 1,000 ppm. However, the response at lower concentration may be lower than some work such as ZnO*/*PANI hybrid [23] and PANI/TiO_2_ nanocomposite thin films [21].

**Figure 8:**
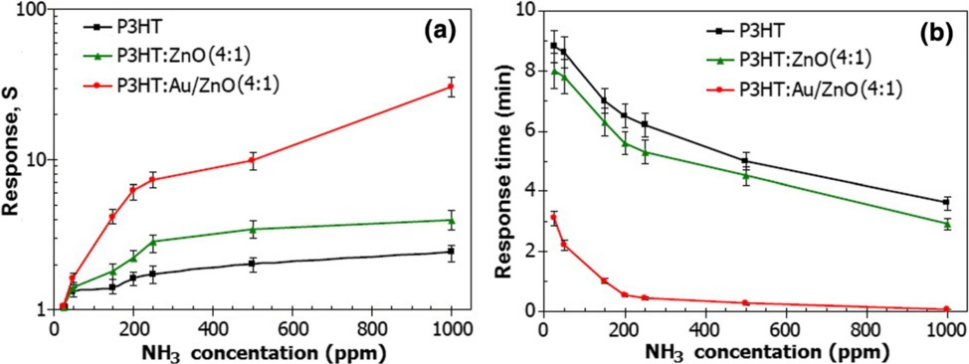
**Sensor response and response time. (a)** Sensor response. **(b)** Response time versus NH_3_ concentration (25 to 1,000 ppm) of P3HT:unloaded ZnO NPs (4:1), P3HT:1.00 mol% Au/ZnO NPs (4:1), and pure P3HT sensors at room temperature.

The sensor characteristics are then analyzed in terms of sensor response and response time. The sensor response (*S*) is determined from the electrical resistance change of P3HT:1.00 mol% Au/ZnO NPs sensors upon exposure to target gas using the following relation: *S* = *R*_gas_/*R*_air_, where *R*_gas_ and *R*_air_ are the stable electrical resistance of a sensor upon exposure to NH_3_ and the initial resistance in air, respectively. The response time is defined as the time needed for a sensor to attain 90% of maximum change in resistance upon exposure to a test gas. The calculated sensor response and response time of optimal sensors with 4:1 of P3HT:1.00 mol% Au/ZnO NPs are shown in Figure 8. Apparently, the sensor response to NH_3_ gas monotonically increases upon exposure with increasing NH_3_ concentration from 25 to 1,000 ppm. At 1,000 ppm, the composite sensor prepared with the 4:1 ratio exhibits the highest NH_3_ response of 32 and a short response time of 4.2 s. Thus, 1.00 mol% Au/ZnO NPs:P3HT composite sensors offer excellent NH_3_ sensing performances with high response, short response time, and room-temperature operation. It should be noted that *y*-error bars of all data correspond to the statistical spread from five sensors of each composition with five evaluations. The statistical results show that fabricated composite sensors offer good repeatability and reproducibility with maximum variation of less than 20%.

The enhanced gas sensing response of the P3HT:1.00 mol% Au/ZnO NPs (4:1) composite sensor may be attributed to the high specific surface area of P3HT surface-coated on granular 1.00 mol% Au/ZnO, which enhances gas adsorption and interaction at the interface [13,21,36]. In order to distinguish the roles of ZnO and gold nanoparticles, the NH_3_ sensing performances of P3HT:ZnO loaded with Au (4:1) are compared with those of P3HT:unloaded ZnO (4:1) and pure P3HT as also demonstrated in Figure 8. It can be seen that the response of the P3HT sensor is only slightly improved by the addition of unloaded ZnO at the mixing ratio of 4:1, while Au addition by loading on ZnO NPs leads to significant increase of NH_3_ response by almost an order of magnitude. In addition, the response time is also substantially reduced to a few minutes or seconds, while ZnO addition does not notably decrease the response time. Thus, Au plays a much more important role than ZnO NPs in enhancing NH_3_ response of the composite sensor. Moreover, it was found from our preliminary study that NH_3_ response of the P3HT:Au-loaded ZnO film increased monotonically as Au loading level increased from 0 to 1.00 mol%. Thus, if Au content increased further, the NH_3_ response should increase to an optimal point and then reduce due to particle aggregation. Further study will be conducted to determine the ultimate optimal Au loading level of the P3HT:flame-made Au-loaded ZnO film for NH_3_ sensing and fully reported elsewhere.

The gas sensing mechanism for the composite sensors may be explained on the basis of interactions between the sensing film and adsorbed gas. For pure P3HT, it has been proposed that NH_3_ can adsorb and donate a lone pair of its electrons to the pentagonal sulfur ring in the P3HT structure [22]. Electrons will recombine with existing holes in the p-type P3HT, leading to a resistance increase in agreement with the observed NH_3_ response. By adding unloaded ZnO NPs, the response is enhanced by a factor of approximately 1.5. This could reasonably be explained by the increase of specific surface area for gas interaction of the composite film by ZnO NPs. From the FE-SEM image in Figure 5, ZnO NP addition results in considerable increase of film porosity and hence the surface area. However, the magnitude of this increase cannot be quantitatively confirmed as BET measurement, which is currently the only quantitative analysis method of specific surface area, of the film surface is presently not available because the standard BET method requires a large amount of material (>0.3 g) which is difficult to prepare in the form of film and the presence of substrate will considerably complicate nitrogen adsorption evaluation. Thus, the specific surface area of the composite film can only be inferred from BET data of the corresponding powder and SEM images.

**Figure 9:**
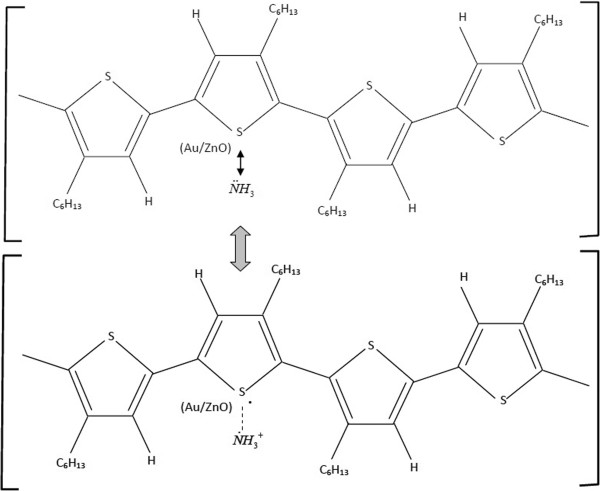
**Proposed model for catalytic effect of Au/ZnO NPs in P3HT:Au/ZnO sensors on NH**_**3 **_**sensing.**

With Au loading, the response is enhanced much more drastically than ZnO NPs and the response also increases with increasing Au loading level from 0 to 1.00 mol%. Considering the effect of surface area change, the BET specific surface area of ZnO NPs is found to increase from 86.3 to 100 m^2^ g^-1^ with 1.00 mol% Au loading (see the ‘Particles and sensing film properties’ section). This corresponds to the 15.9% increase, and the influence of specific surface area alone cannot explain the observed large response enhancement by Au loading on ZnO. From the results, Au loading on ZnO increases not only the response magnitude but also the response rate substantially. Thus, the most plausible mechanism for such enhancement should be the catalytic effect of Au on ZnO NPs. Figure 9 depicts our proposed model for the catalytic effect of Au/ZnO NPs based on a P3HT-ammonia interaction mechanism reported recently [17]. In this model, it is assumed that Au/ZnO NPs located around sulfur atoms in the pentagonal rings of P3HT catalyze the reaction, causing more NH_3_ molecules to give lone-pair electrons and form the weak binding. The probability for Au/ZnO NPs should be high since gold and sulfur have rather strong binding affinity. To obtain effective catalyst activity, Au NPs should be uniformly dispersed throughout the P3HT matrix. Thus, Au plays the main role in enhancing NH_3_ interaction and response with P3HT, while the role of ZnO NPs is the supports that help formation and dispersion of ultrafine Au nanoparticles. However, when only 1.00 mol% Au/ZnO is used, there is no response since Au catalyzes the reaction between NH_3_ and P3HT.

For the effect of composite composition, the results show that 4:1 of P3HT:1.00 mol% Au/ZnO NPs, which is the composite with the lowest 1.00 mol% Au/ZnO NP content, offers the highest NH_3_ sensing enhancement and the enhancement decreases with increasing Au/ZnO NP content. A plausible explanation is that 1.00 mol% Au/ZnO NPs are well dispersed in the P3HT matrix at this low concentration, yielding a homogeneous distribution of Au/ZnO NPs throughout the layer and enabling effective catalytic interaction with NH_3_ gas. In addition, the well-dispersed structure should be highly porous and exhibit large surface area for gas interaction. As the content of 1.00 mol% Au/ZnO NPs increases, the Au/ZnO NPs may begin to agglomerate resulting in less homogenous formation of Au/ZnO NPs and less porous composite structures, which lead to low NH_3_ sensing response. Based on the current results, NH_3_ sensing properties of the composite film may be further improved by optimizing the structure/composition of the Au loading material as well as metal oxide support to maximize the catalytic effect and by adding intercalating nanomaterials with different dimensionalities (i.e., 2D graphene, 1D metal oxide nanowire, 1D carbon nanotubes, etc.) to reduce particle agglomeration and increase effective surface area. Moreover, new catalysts based on the composite of Au and other catalytic materials should be explored to further improve the catalytic effect.

**Figure 10:**
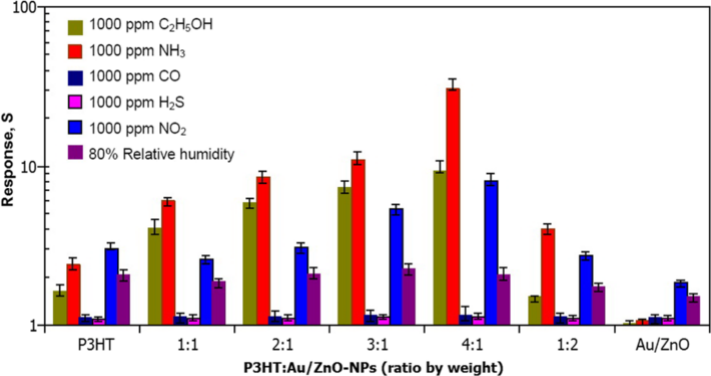
**Relative response.** The relative response to NH_3_ (1,000 ppm), C_2_H_5_OH (1,000 ppm), CO (1,000 ppm), H_2_S (1,000 ppm), NO_2_ (1,000 ppm), and H_2_O (80% RH) of sensors with difference ratio of P3HT:1.00 mol% Au/ZnO NPs (1:0, 1:1, 2:1, 3:1, 4:1, 1:2, and 0:1).

Selectivity can be defined as the ability of a sensor to respond to a target gas in the presence of other interfering gases [12]. The NH_3_ sensing selectivity of composite sensors is characterized toward various reducing and oxidizing gases including ethanol (C_2_H_5_OH), carbon monoxide (CO), hydrogen sulfide (H_2_S), and nitrogen dioxide (NO_2_) at 1,000 ppm and room temperature as shown in Figure 10. In addition, the effect of water vapor is included at 80% RH. It is evident that the composite sensor of P3HT:1.00 mol% Au/ZnO NPs (4:1) exhibits a relatively high response of 32 to 1,000 ppm of NH_3_ while the response to 1,000 ppm of C_2_H_5_OH and NO_2_ is relatively low (approximately 9 and approximately 8, respectively), and those of 1,000 ppm of CO and 1,000 ppm of H_2_S are almost negligible. Additionally, the optimal sensor exhibits a quite low response of approximately 2.2 to a high relative humidity of 80%. For P3HT and other composite combinations, the response to 1,000 ppm of NH_3_ is not much higher than that to C_2_H_5_OH, NO_2_, and humidity. The results indicate that P3HT:1.00 mol% Au/ZnO NPs also has better selectivity to NH_3_ against C_2_H_5_OH, CO, H_2_S, NO_2_, and humidity than other sensors. Therefore, the composite sensor of P3HT:1.00 mol% Au/ZnO NPs (4:1) can be used for selective detection of NH_3_.

Lastly, the stability of P3HT-based sensors has been evaluated by monitoring the response change over 30 days. It was found that the pure P3HT sensor had an average response reduction of around 4.8%/day, while P3HT with 1.00 mol% Au/ZnO NPs and unloaded ZnO NPs at different ratios exhibits slightly lower average response reduction in the range of 4.2% to 4.6%/day. It is not conclusive whether ZnO NPs help improve the stability of P3HT sensors. Nevertheless, it is seen that the ZnO NPs:P3HT sensor has fair medium-term stability, which is relatively high compared with other conductive polymers.

## Conclusions

In conclusion, novel composite P3HT:1.00 mol% Au/ZnO NPs films have been systemically studied for NH_3_ sensing applications at room temperature. The unloaded ZnO and ZnO NPs loaded with Au (1.00 mol%) were produced by a single-step FSP technique. The particle analyses using XRD, HR-TEM, and BET indicated that ZnO NPs were highly crystalline with a typical hexagonal structure of ZnO, and ultrafine Au NPs with 1 to 2 nm in diameter were formed around ZnO NPs. Composite P3HT:1.00 mol% Au/ZnO NPs films with different compositions were prepared by solution mixing and casting. Film characterizations by XRD and FE-SEM confirmed the presence of P3HT/ZnO phases and porous nanoparticle structures in the composite thick film. The gas sensing results showed that the inclusion of 1.00 mol% Au/ZnO NPs at a low content provided significant NH_3_ sensing enhancement. In particular, the P3HT:1.00 mol% Au/ZnO NPs composite film with the ratio of 4:1 exhibited the best NH_3_ sensing performances with a high sensor response of approximately 32 and short response time within a minute to 1,000 ppm of NH_3_ at a room temperature. In addition, the optimal composite film exhibited higher NH_3_ selectivity against C_2_H_5_OH, CO, H_2_S, NO_2_, and H_2_O than other composites as well as P3HT and 1.00 mol% Au/ZnO NPs. The observed composite gas sensing behaviors were explained based on the increased specific surface area by porous blended nanoparticle structure and catalytic effect of Au/ZnO NPs. From overall results, the P3HT:1.00 mol% Au/ZnO NPs composite sensor is a highly promising candidate for the efficient detection of NH_3_ at room temperature.

## Abbreviations

AFM: atomic force microscopy; BET: Brunauer-Emmett-Teller; EDX: energy-dispersive X-ray; FE-SEM: field-emission scanning electron microscopy; FSP: flame spray pyrolysis; HR-TEM: high-resolution transmission electron microscopy; NPs: nanoparticles; P3HT: poly (3-hexylthiophene); SSA_BET_: specific surface area; XRD: X-ray diffraction.

## Competing interests

The authors declare that they have no competing interests.

## Authors’ contributions

VK carried out the experiments, acquired the original data, participated in the sequence alignment, and drafted the manuscript. AW and SP have made substantial contributions to the conception and design for this article. AT read the final manuscript. All the authors read and approved the final manuscript.
